# Differential diagnosis of pulmonary nodules and prediction of invasive adenocarcinoma using extracellular vesicle DNA

**DOI:** 10.1002/ctm2.1582

**Published:** 2024-02-12

**Authors:** Chenghu Song, Yifeng Sun, Yundi Chen, Yihang Shen, Haozhi Lei, Wenjun Mao, Jing Wang, Yuan Wan

**Affiliations:** ^1^ Department of Cardiothoracic Surgery The Affiliated Wuxi People's Hospital of Nanjing Medical University Wuxi Jiangsu China; ^2^ Department of Biomedical Engineering The Pq Laboratory of BiomeDx/Rx Binghamton University Binghamton New York USA; ^3^ Department of Surgery Ulm University Hospital, Ulm University Ulm Germany; ^4^ Department of Surgery Heidelberg University Hospital Heidelberg University Heidelberg Germany; ^5^ Department of Computational Biology Carnegie Mellon University Pittsburgh Pennsylvania USA; ^6^ Institute of Molecular Medicine, Renji Hospital, School of Medicine, Shanghai Jiao Tong University Shanghai China; ^7^ Yizheng Hospital of Nanjing Drum Tower Hospital Group Yizheng Jiangsu China; ^8^ Department of Hematology Nanjing Drum Tower Hospital, Affiliated Hospital of Medical School, Nanjing University Nanjing Jiangsu China

To the Editor:

1

The differential diagnosis of pulmonary nodules (PN) poses significant challenges as they can be observed in both benign and malignant aetiology.^1^, ^2^, ^3^ Computed tomography (CT)‐based differentiation between malignant PN (MPN) and benign PN (BPN) is moderately effective.^4^, ^5^ Tissue biopsy, while facilitating diagnosis, is hindered by small size and sampling bias.^6^, ^7^ Here, using identified high‐frequency mutations from MPN, we developed a compact 21‐gene panel for liquid biopsy and a diagnostic nomogram model to predict the risk of invasive adenocarcinoma (IAC).

A total of 79 patients with MPN confirmed by CT imaging and pathological examination were enrolled. No significant differences were observed in age, sex or smoking status among groups (Table S1). The genomic analysis of MPN tissue DNA was performed (Figure S1A). The median value of variants per sample was 10 (Figure S1B). Top 20 mutated genes were disclosed (Figure S1C). The data revealed no significant differences in tumour mutation burden (TMB) or mutation points across adenocarcinoma in situ (AIS), minimally invasive adenocarcinoma (MIA) and IAC due to a small sample size (Figure S1D). Significant pairs of co‐occurring driver genes and mutually exclusive genes were observed in all samples, AIS, MIA and IAC, respectively (Figure S2A–D). Subsequently, we found that EGFR, TP53 and NOTCH3 are prevalent drivers irrespective of sex, while BLM, KRAS and MSH3 showed sex‐specific differences (Figure S3A,B). Due to the similar composition of these groups, the top mutated genes in nonsmokers were identical to those in female, and the top mutated genes in smokers were identical to those in male. Contrastingly, the top mutated genes in nine male nonsmokers were largely distinct. In addition, genomic analysis of nonsmokers and smokers with MPN mirrored overall population results. Eight smoking‐related unfavourable mutant genes were identified, along with nine mutant genes unaffected by sex and smoking status, indicating pervasive driver gene effects (Figure S3C). Moreover, we observed that smokers with MPN are more prone to developing mutations in anti‐oncogenes, while nonsmokers with MPN tend to have mutations in oncogenes.^8^, ^9^ This reflects the distinct patterns in tumorigenesis between the two groups.

Next, we divided the IAC data into training and testing sets at a 2:1 ratio (Figure 1A,B). Fisher's exact test confirmed no significant difference in gene mutation frequency between the two. We recorded the prevalent mutations identified by the 618‐gene panel in the 79 patients. Through analysis of various gene combinations, we fine‐tuned a 21‐gene panel that can identify 77 out of 79 patients. Enriched signal pathways (Figure S4A) and significant gene ontologies (Figure S4B) were identified in this gene set. Significantly mutated pathways were more closely related to Wnt‐beta catenin signalling, E2F targets pathway and Notch signalling. Previous studies have reported similar findings, which substantiates the findings of our study. Meanwhile, we developed a nomogram model to predict likelihood of IAC occurrence using logistic regression analysis (Table S2). Currently, IAC diagnosis relies on postoperative histological examination, which may be inaccurate due to biased sampling and imprecise inspection, especially if subtle lesions are missed.^10^ Our model aims to identify IAC prior to surgery. A higher score prompts the surgeon to consider IAC occurrence, perform lymph node removal, and closely monitor for recurrence. We identified that PN diameter and TMB of the 21 genes significantly predict the likelihood of IAC occurrence. The calibration curve showed good agreement in this cohort (Figure 1C). The concordance index was .849, showing excellent discrimination, reconfirmed at .809 through bootstrapping validation. The receiver operating characteristic (ROC) demonstrated good predictive accuracy (Figure 1D).Decision curve analysis (DCA) showed that if the threshold probability was greater than 4%, the nomogram provides better diagnosis probability (Figure 1E).

**FIGURE 1:**
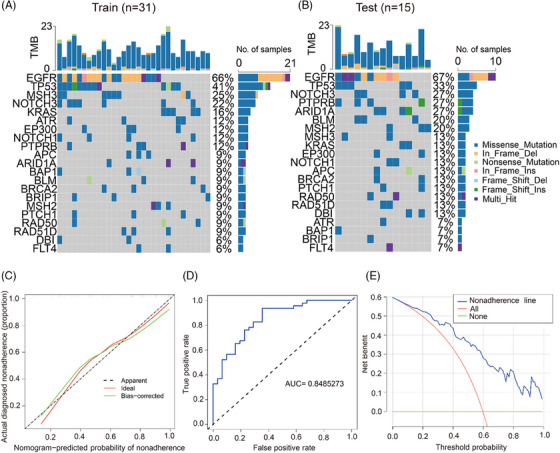
Significantly mutated genes and nomogram model for predicting invasive adenocarcinoma (IAC) risk. (A and B) The mutation information of each gene in the training and testing sets. (C) Calibration plot. The prediction results (green solid line) were closer fit to the ideal model (diagonal line). (D) ROC that shows a good predictive accuracy. (E) DCA for the diagnostic nomogram. The blue solid line represents the IAC diagnostic nomogram. The red solid line represents the assumption that all patients are not diagnosed as IAC. Thin green solid line represents the assumption that no patients are IAC.

Finally, 18 MPN and two BPN were enrolled for validation (Table S3). Preoperative circulating extracellular vesicles (EVs) were isolated, followed by electron microscope analysis (Figure S5A), size measurement (Figure S5B) and Western blot analysis of CD9 (Figure S5C). Subsequently, EVs, tumour tissue and normal adjacent tissue samples were analysed using the 21‐gene panel, which provides an overall coverage of 99.7%. Over approximately 92% of reads were identified to index. Mutations were detected in 19 genes in tissue DNA and in 17 genes in EV DNA (Figure 2A). In contrast, only EP300, BPN and TP53 mutations were detected in BPN tissue and EV DNA. Missense mutations predominated in MPN and BPN (Figure 2B). Subsequent analysis was conducted on 16 MPN and two BPN with paired tissue and EV DNA. Significant difference in TMB, mutation type and number of mutated gene were found between MPN and BPN in both tissue and EV DNA (Figure 2C). TMB displayed an increasing trend from T1a to T1c, showing promise in MPN staging (Figure 2D). A paired bar chart of the mutation gene count for the 18 samples was further generated (Figure 2E), and the average concordance of gene mutations was 75.3% in MPN and 50% in BPN (Figure 2F). In brief, the 21‐gene panel can identify MPN using EV DNA. Calculated in 20 patients, the average risk score for IAC occurrence in MPN tissue and EV samples was 93.98 ± 25.07 and 89.60 ± 23.3, respectively, with a predicted risk of .72 ± .26. In contrast, the average risk score for BPN tissue and EV samples was 57.11 ± 27.98, corresponding to a predicted risk of .34 ± .33 (Figure 3A). The findings align with existing clinicopathological data. No significant difference across stages T1a, T1b and T1c was observed (Figure 3B). In two patients without tissue samples, IAC occurrence risk, determined from EV DNA TMB, was 60.64 and 71.5, corresponding to risks of .34 and .5, respectively. The lower score in one IAC‐diagnosed patient (57 years, T1b, 1.1 cm) was mainly attributed to low TMB. Moreover, we found a strong correlation between the TMB score of tissue DNA and EV DNA samples in MPN patients (Figure 3C).

**FIGURE 2:**
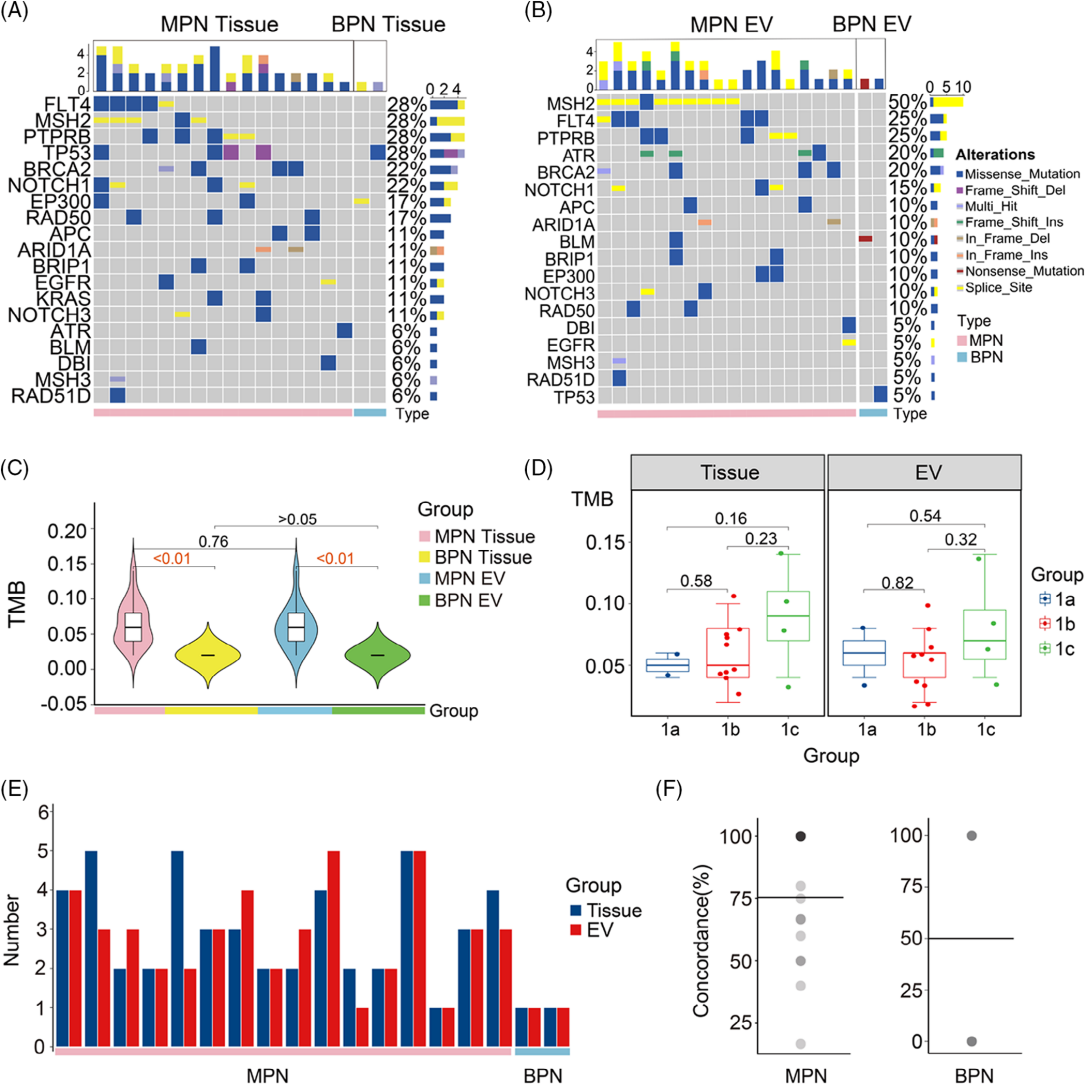
Validation of the 21‐gene panel with tumour tissue DNA and extracellular vesicle (EV) DNA. (A and B) The waterfall plots demonstrate the mutation information of each gene in malignant pulmonary nodules (MPN) and benign pulmonary nodules (BPN) samples, respectively. (C) Tumour mutation burden (TMB) in tissue DNA and EV DNA derived from 18 samples, respectively. (D) TMB among stage T1a, T1b and T1c in tissue DNA and EV DNA, respectively. (E) Paired bar chart of the mutation gene count for the 18 samples. (F) The average concordance of gene mutations in MPN and BPN samples, respectively.

**FIGURE 3:**
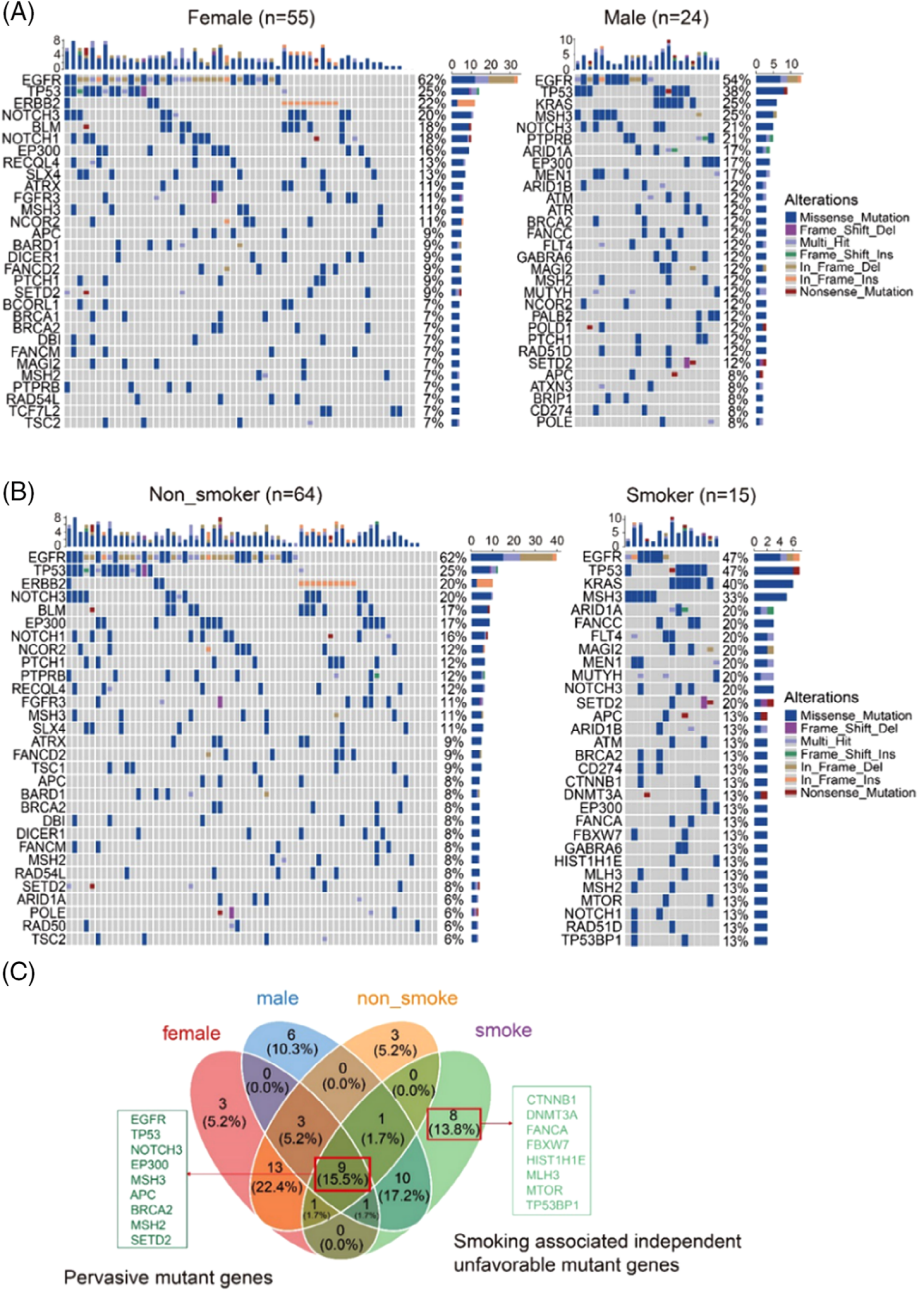
Validation of the diagnostic nomogram model with paired tissue DNA and extracellular vesicle (EV) DNA samples. (A) Predicting points using the diagnostic nomogram model for malignant pulmonary nodules (MPN) and benign pulmonary nodules (BPN) samples based on tissue DNA and EV DNA, respectively. (B) Predicting points among stage T1a, T1b and T1c using tissue DNA and EV DNA, respectively. (C) Conducting correlation analysis of tumour mutation burden (TMB) scores between tissue DNA and EV DNA using the Pearson method.

In summary, this study delves into the genomic landscape and mutational signatures in MPN. The 21‐gene panel with EV DNA can identify MPN in a short turnaround time, while remaining affordable for patients. The nomogram can evaluate the risk of IAC, thus providing valuable insights for healthcare decision‐making. The diagnostic strategy holds promise for clinical translation pending a large‐scale study.

## AUTHOR CONTRIBUTIONS

The project was designed by Yuan Wan, Wenjun Mao and Jing Wang. Chenghu Song, Yifeng Sun, Yundi Chen, Yihang Shen and Haozhi Lei performed the experiments, collected and analysed the data. All authors contributed to the writing of the manuscript, discussed the results and implications, and edited the paper at all stages.

## CONFLICT OF INTEREST STATEMENT

The authors declare that they have no known competing financial interests or personal relationships that could have appeared to influence the work reported in this paper.

## FUNDING INFORMATION

National Cancer Institute, Grant Numbers: R37CA255948, R01CA230339.

## ETHICS STATEMENT

All patients in this study provided written informed consent. The study protocol was conducted in accordance with the principles of the Declaration of Helsinki and was approved by the Research Ethics Committee of Wuxi People's Hospital affiliated to Nanjing Medical University (No. HS2019014).

## Supporting information

Supporting Information

## Data Availability

All relevant data of this study are presented in the paper and Supporting Information file. Sequencing data were deposited for public access (https://www.ncbi.nlm.nih.gov/bioproject/PRJNA978942 and https://www.ncbi.nlm.nih.gov/sra/PRJNA1006451).
