# Supplementary material for: Differential diagnosis of pulmonary nodules and prediction of invasive adenocarcinoma using extracellular vesicle DNA
Source: Clin Transl Med. 2024 Feb 12;14(2):e1582. doi: 10.1002/ctm2.1582 (PMC10859785; doi:10.1002/ctm2.1582)
Supplement: Supplementary file 1 — Supporting Information [file CTM2-14-e1582-s001.docx]

**Supplementary Information for**

**Liquid biopsy of pulmonary nodules with extracellular vesicle DNA**

by Song *et al.*

**Materials and Methods**

**Patients**

Patients diagnosed with PN were recruited between June 2020 and April 2022. A written informed consent was provided. The study protocol was conducted in accordance with the principles of the Declaration of Helsinki and was approved by the Research Ethics Committee of Wuxi People’s Hospital affiliated to Nanjing Medical University (No. HS2019014).

**Isolation and characterization of extracellular vesicles**

Approximately 10-ml blood samples were drawn into 10-ml EDTA (K2) tubes (BD). After centrifugation at 2,500 g for 15 min plasma was collected. The plasma samples were further centrifuged at 16,500g for 20 min at 4 °C and filtered using a 0.22-μm pore filter. Subsequently, the filtrated plasma samples were ultracentrifuged at 100,000 g at 4 °C for 4 h. The EV pellets were suspended in 100 μl of PBS. Approximately, 5 μl of EV samples was placed on 200 mesh Formvar-coated copper grids and incubated for 5 min at room temperature. Excess samples were blotted with filter paper followed by negative staining with 1% uranyl acetate for 1 min. Samples were examined in a FEI Tecnai transmission electron microscope at an accelerating voltage of 100 kV. Surplus EV samples treated with lysis buffer containing 8 M urea/2.5% SDS, 5 μg/ml leupeptin, 1 μg/ml pepstatin, and 1 mM phenylmethylsulfonyl fluoride buffer. Samples were analyzed using acrylamide gels and then transferred onto PVDF membranes. The protein blot was blocked for 1 h at room temperature with 5% non-fat dry milk in PBS/0.05% Tween and incubated overnight at 4 °C with HRP-labeled anti-CD9 antibody (Santa Cruz, sc-13118). After washing with PBS/0.05% Tween 20 for 10 min thrice, blots were developed with Pierce10. EV size distribution was measured with Nanosight NS300 according to manufacturer's instructions.

**Histological examination and sequencing**

Histologic type was examined by two pathologists, and tumor purity was assessed prior to DNA extraction. Tissue and EV DNA were isolated using the DNeasy kit (Qiagen 69504) and QIAamp DNA micro kit (Qiagen 56304), respectively. A 618-gene panel (Agilent) was used to analyze tissue DNA followed by analyses including mutational signatures, tumor mutation burden (TMB), co-occurrence and mutual exclusivity (COME), gene ontology (GO), and pathway enrichment. Subsequently, at least 250 ng of tissue and at least 30 ng of paired EV DNA from 20 patients with PN was analyzed with a 21-gene panel (TE-93316425, Twist) on an Illumina NextSeq 2000.

**Diagnostic nomogram model**

To predict the relationship between various predictors and IAC occurrence, multivariable logistic regression analysis was performed using the primary cohort. The logistic regression analysis that incorporates factors such as gender, smoking status, age, tumor diameter, and tumor mutation burden associated with the 21-gene panel.

**Statistical Analysis**

The Clinical and pathological parameters of this cohort were evaluated by the Kruskal-Wallis test for continuous variables, and chi-square analysis or Fisher’s exact test for categorical variables. The MutSigCV algorithm was applied to assess significant somatic mutations for each gene. Mutational signatures associated with MPN were identified using the non-negative matrix factorization algorithm. The Cancer Progression Inference Algorithm was used to test these mutational signatures. Statistical analyses and visualization were performed using R software. The “glmnet”, “rms”, “Hmisc”, “rmda” R packages were used to construct and evaluate the diagnostic nomogram model. GO and pathway enrichment analyses were conducted on the Enrichr. A p-value of <0.05 was considered statistically significant.

**Table S1.** Clinicopathologic characteristics of 79 patients with MPN

|  | **Total (n=79)** | **AIS (n=11)** | **MIA (n=20)** | **IAC (n=48)** | **p value** |
| --- | --- | --- | --- | --- | --- |
| **Age (yrs)** | 58.8 (29-79) | 54.5 (35-76) | 55.7 (29-79) | 61.0 (29-76) | 0.0822 |
| **Sex** |  |  |  |  |  |
| male | 24 (30.4%) | 2 (18.2%) | 5 (25%) | 17 (35.4%) | 0.5676 |
| female | 55 (69.6%) | 9 (81.8%) | 15 (75%) | 31 (64.6%) |  |
| **Smoking status** |  |  |  |  | 0.6196 |
| nonsmoker | 15 (19%) | 1 (9.1%) | 2 (10%) | 12 (25%) |  |
| smoker | 64 (81%) | 10 (90.9%) | 10 (90%) | 36 (75%) |  |
| **Tumor size (mm)** | 15.4 (6-40) | 7.9 (6-12) | 12.7 (7-22) | 18.3 (7-40) | <0.01 |
| **Tumor site** |  |  |  |  | 0.9230 |
| upper right | 35 (44.3%) | 4 (36.4%) | 10 (50%) | 21 (43.8%) |  |
| lower right | 14 (17.7%) | 3 (27.3%) | 4 (20%) | 7 (14.6) |  |
| middle right | 4 (5.1%) | 1 (9.1%) | 1 (5%) | 2 (4.2%) |  |
| upper left | 18 (22.8%) | 2 (18.2) | 3 (15%) | 13 (27.1%) |  |
| lower left | 8 (10.1%) | 1 (9.1%) | 2 (10%) | 5 (10.4%) |  |
| **Tumor staging** |  |  |  |  | <0.01 |
| 0 | 11 (13.9%) | 11(100%) | 0 | 0 |  |
| Ia1 | 25 (31.6%) | 0 | 20 (100%) | 5 (10.4%) |  |
| Ia2 | 26 (32.9%) | 0 | 0 | 26 (54.2%) |  |
| Ia3 | 5 (6.3%) | 0 | 0 | 5 (10.4%) |  |
| Ib | 12 (15.2%) | 0 | 0 | 12 (25%) |  |

**Table S2.** Multivariate logistic regression analysis

|  | **MPN** | **BPN** | ***p* value** |
| --- | --- | --- | --- |
| **Gender (No.)** |  |  |  |
| **Female** | 30 | 24 | 0.72583 |
| **Male** | 16 | 7 |  |
| **Smoking (No.)** | 11 | 3 | 0.73761 |
| **Age (Mean±SD, Yr)** | 61±9 | 55±13 | 0.10995 |
| **Pulmonary nodule**  **diameter (Mean±SD, cm)** | 1.8±0.8 | 1.0±0.4 | 0.00795* |
| **21-gene TMB/Mb (Mean±SD)** | 0.08±0.04 | 0.04±0.03 | 0.01380* |

**Table Description:** We conducted a multivariate logistic regression analysis and identified that both the pulmonary nodule diameter and the Tumor Mutational Burden (TMB) of 21 genes significantly predict the likelihood of IAC (Invasive Adenocarcinoma) occurrence.

**Table S3**. Clinicopathological information of PNs investigated in this study (in the cohort of 79 patients, light blue, medium blue, and dark blue represents AIS, MIA, and IAC; in the cohort of 21 patients, light blue, medium blue, and dark blue represents T1a, T1b, and T1c;).

| 79 patients analyzed by a 618-gene panel | | | | | | |
| --- | --- | --- | --- | --- | --- | --- |
| **Sex** | **Stage** |  | **Smoking** | **Age** | **Location** | **Size (cm)** |
| M | AIS | Tis | Y | 75 | Upper Right | 0.8 |
| F | AIS | Tis | N | 69 | Lower Right | 0.7 |
| F | AIS | Tis | N | 59 | Lower Right | 0.9 |
| F | AIS | Tis | N | 46 | Lower Left | 0.7 |
| F | AIS | Tis | N | 48 | Upper Right | 1.2 |
| F | AIS | Tis | N | 76 | Upper Right | 0.7 |
| F | AIS | Tis | N | 35 | Upper Right | 0.6 |
| F | AIS | Tis | N | 43 | Lower Right | 0.7 |
| F | AIS | Tis | N | 37 | Upper Left | 0.6 |
| M | AIS | Tis | N | 59 | Middle Right | 0.8 |
| F | AIS | Tis | N | 53 | Upper Left | 1 |
| F | MIA Ia2 | T1b | N | 29 | Lower Right | 1.3 |
| F | MIA Ia2 | T1b | N | 56 | Upper Right | 1.2 |
| F | MIA Ia2 | T1b | N | 46 | Upper Right | 1.2 |
| M | MIA Ia2 | T1b | N | 65 | Upper Right | 1.5 |
| F | MIA Ia2 | T1b | N | 51 | Upper Right | 1.2 |
| F | MIA Ia2 | T1b | N | 79 | Upper Right | 2 |
| F | MIA Ia2 | T1b | N | 61 | Upper Left | 1.5 |
| M | MIA Ia2 | T1b | Y | 70 | Upper Right | 1.2 |
| F | MIA Ia1 | T1a | N | 46 | Lower Right | 1 |
| F | MIA Ia3 | T1c | N | 59 | Lower Right | 2.2 |
| F | MIA Ia2 | T1b | N | 43 | Upper Right | 1.4 |
| F | MIA Ia1 | T1a | N | 31 | Lower Left | 0.8 |
| F | MIA Ia1 | T1a | N | 54 | Middle Right | 0.7 |
| M | MIA Ia2 | T1b | N | 55 | Upper Right | 1.4 |
| F | MIA Ia1 | T1a | N | 69 | Lower Right | 1 |
| M | MIA Ia1 | T1a | N | 53 | Lower Left | 0.8 |
| M | MIA Ia2 | T1b | Y | 65 | Upper Left | 1.5 |
| F | MIA Ia1 | T1a | N | 57 | Upper Right | 0.9 |
| F | MIA Ia2 | T1b | N | 64 | Upper Left | 1.7 |
| F | MIA Ia1 | T1a | N | 61 | Upper Right | 0.8 |
| M | IAC Ia2 | T1b | Y | 69 | Lower Left | 1.7 |
| M | IAC Ia3 | T1c | Y | 63 | Upper Left | 2.5 |
| F | IAC Ia1 | T1a | N | 60 | Upper Left | 1 |
| F | IAC Ia2 | T1b | N | 57 | Upper Right | 1.3 |
| M | IAC Ia2 | T1b | Y | 48 | Lower Left | 1.8 |
| M | IAC Ia2 | T1b | N | 71 | Upper Left | 2 |
| F | IAC Ia2 | T1b | N | 71 | Upper Right | 1.4 |
| F | IAC Ia2 | T1b | N | 65 | Upper Right | 2 |
| F | IAC Ia3 | T1c | N | 67 | Lower Left | 3 |
| F | IAC Ia2 | T1b | N | 58 | Upper Left | 1.5 |
| F | IAC Ia3 | T1c | N | 61 | Upper Left | 2.2 |
| F | IAC Ia2 | T1b | N | 67 | Lower Left | 1.2 |
| F | IAC Ia2 | T1b | N | 62 | Upper Right | 2 |
| F | IAC Ia2 | T1b | N | 54 | Upper Right | 1.2 |
| M | IAC Ia2 | T1b | N | 50 | Upper Right | 1.8 |
| M | IAC Ia3 | T1c | Y | 63 | Lower Right | 2.8 |
| F | IAC Ia2 | T1b | N | 51 | Upper Right | 1.2 |
| F | IAC Ia2 | T1b | N | 47 | Middle Right | 1.3 |
| M | IAC Ia2 | T1b | Y | 61 | Upper Left | 1.2 |
| F | IAC Ia2 | T1b | N | 49 | Upper Right | 1.2 |
| M | IAC Ia2 | T1b | Y | 74 | Upper Left | 1.2 |
| F | IAC Ia1 | T1a | N | 59 | Middle Right | 1 |
| F | IAC Ia2 | T1b | N | 67 | Upper Left | 1.2 |
| M | IAC Ia2 | T1b | Y | 69 | Upper Right | 1.2 |
| F | IAC Ia3 | T1c | N | 29 | Upper Left | 2.8 |
| F | IAC Ia2 | T1b | N | 62 | Upper Left | 1.5 |
| M | IAC Ia2 | T1b | Y | 70 | Lower Right | 1.8 |
| M | IAC Ia1 | T1a | N | 52 | Lower Right | 1 |
| F | IAC Ia1 | T1a | N | 65 | Lower Right | 0.8 |
| F | IAC Ia2 | T1b | N | 53 | Upper Right | 1.5 |
| F | IAC Ia2 | T1b | N | 62 | Lower Left | 1.3 |
| M | IAC Ia2 | T1b | Y | 70 | Upper Right | 1.5 |
| M | IAC Ia2 | T1b | N | 51 | Upper Right | 1.2 |
| F | IAC Ia1 | T1a | N | 74 | Lower Right | 0.7 |
| F | IAC Ia2 | T1b | N | 71 | Upper Right | 2 |
| F | IAC Ia2 | T1b | N | 56 | Upper Right | 2 |
| F | IAC Ib | T2 | N | 74 | Upper Right | 1.4 |
| M | IAC Ib | T2 | Y | 59 | Upper Right | 3.3 |
| M | IAC Ib | T2 | N | 52 | Upper Right | 1.5 |
| F | IAC Ib | T2 | N | 72 | Upper Left | 1.2 |
| F | IAC Ib | T2 | N | 75 | Upper Right | 3 |
| M | IAC Ib | T2 | Y | 59 | Upper Right | 3.5 |
| M | IAC Ib | T2 | Y | 62 | Upper Left | 4 |
| F | IAC Ib | T2 | N | 76 | Lower Right | 2.8 |
| F | IAC Ib | T2 | N | 57 | Upper Right | 4 |
| F | IAC Ib | T2 | N | 44 | Upper Left | 2.9 |
| F | IAC Ib | T2 | N | 59 | Lower Right | 1.5 |
| F | IAC Ib | T2 | N | 63 | Upper Right | 1.7 |

| 20 patients analyzed by a 21-gene panel | | | | | |  |
| --- | --- | --- | --- | --- | --- | --- |
| **Sex** | **Stage** | **Smoking** | **Age** | **Location** | **Size (cm)** | **Tissue** |
| F | T1a | Y | 53 | Lower Right | 0.7 |  |
| F | T1a | Y | 70 | Upper Right | 0.6 |  |
| F | T1b | Y | 60 | Upper Left | 0.9 |  |
| M | T1b | Y | 81 | Upper Right | 0.8 |  |
| F | T1b | Y | 53 | Lower Right | 2 |  |
| F | T1b | Y | 57 | Lower Left | 1.1 | N.A. |
| F | T1b | N | 63 | Upper Left | 2.6 |  |
| F | T1b | Y | 60 | Upper Left | 1.8 |  |
| F | T1b | N | 66 | Middle Right | 2.4 |  |
| F | T1b | Y | 51 | Upper Right | 1.2 |  |
| F | T1b | Y | 58 | Lower Right | 1.1 |  |
| M | T1b | Y | 62 | Upper Left | 2.6 |  |
| F | T1b | Y | 61 | Lower Right | 2.7 |  |
| F | T1c | Y | 60 | Upper Right | 1.4 | N.A. |
| M | T1c | Y | 49 | Upper Right | 2.1 |  |
| M | T1c | N | 80 | Upper Right | 1.3 |  |
| F | T1c | Y | 60 | Upper Right | 1.4 |  |
| F | T1c | Y | 78 | Upper Left | 1.3 |  |
|  |  |  |  |  |  |  |
| F | Inflammation | N | 46 | Middle Right | 0.8 |  |
| M | Granuloma | Y | 70 | Lower Right | 1.5 |  |

**
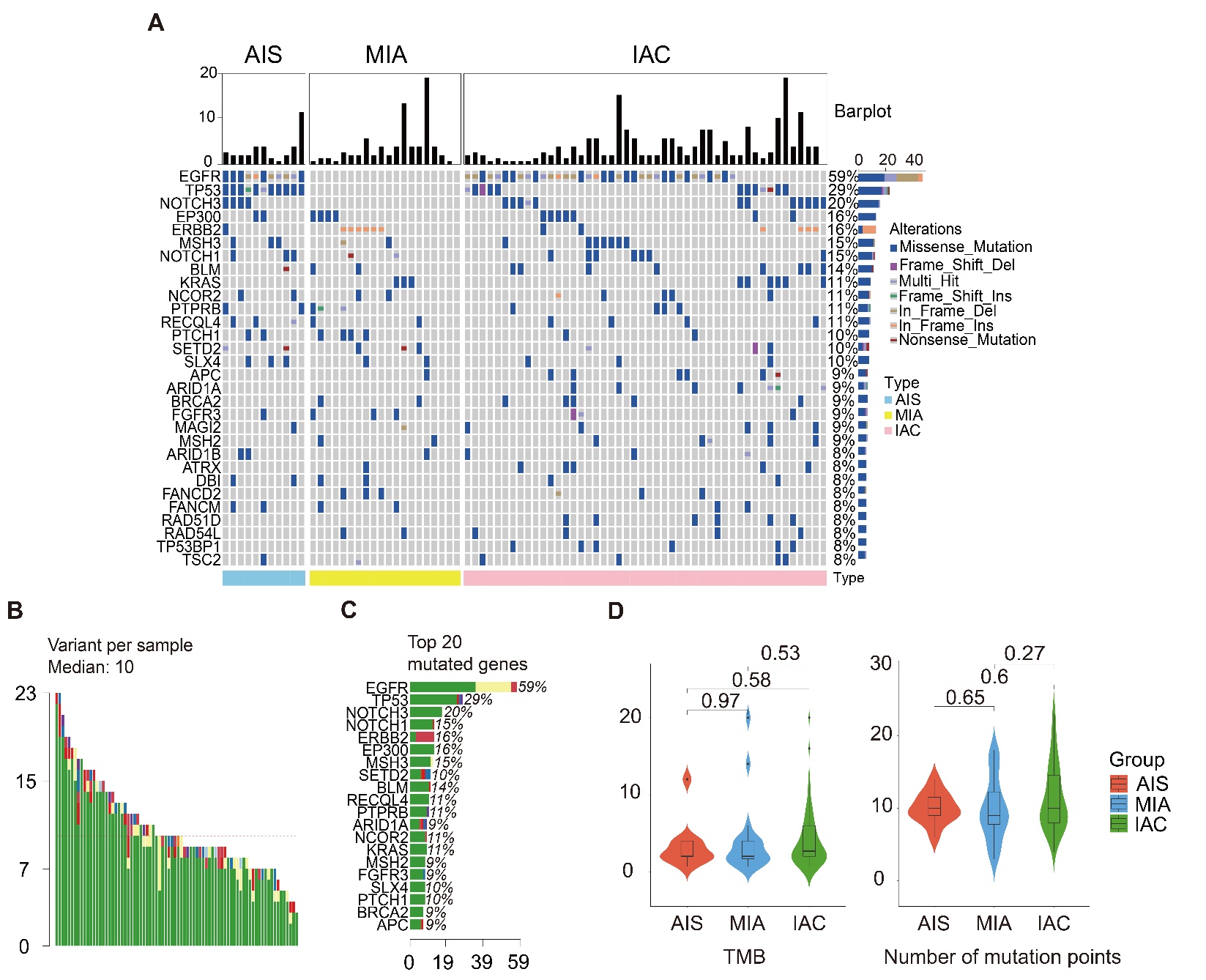
Figure S1**. Genomic landscape of MPN. (A) The waterfall plot shows the top 30 mutated genes in MPN tissue across AIS, MIA, and IAC. (B) Illustration of variants per sample and summary of variant classification. (C) The top 20 mutated genes in MPN. (D) TMB (left) and number of mutation points (right) across AIS, MIA, and IAC.


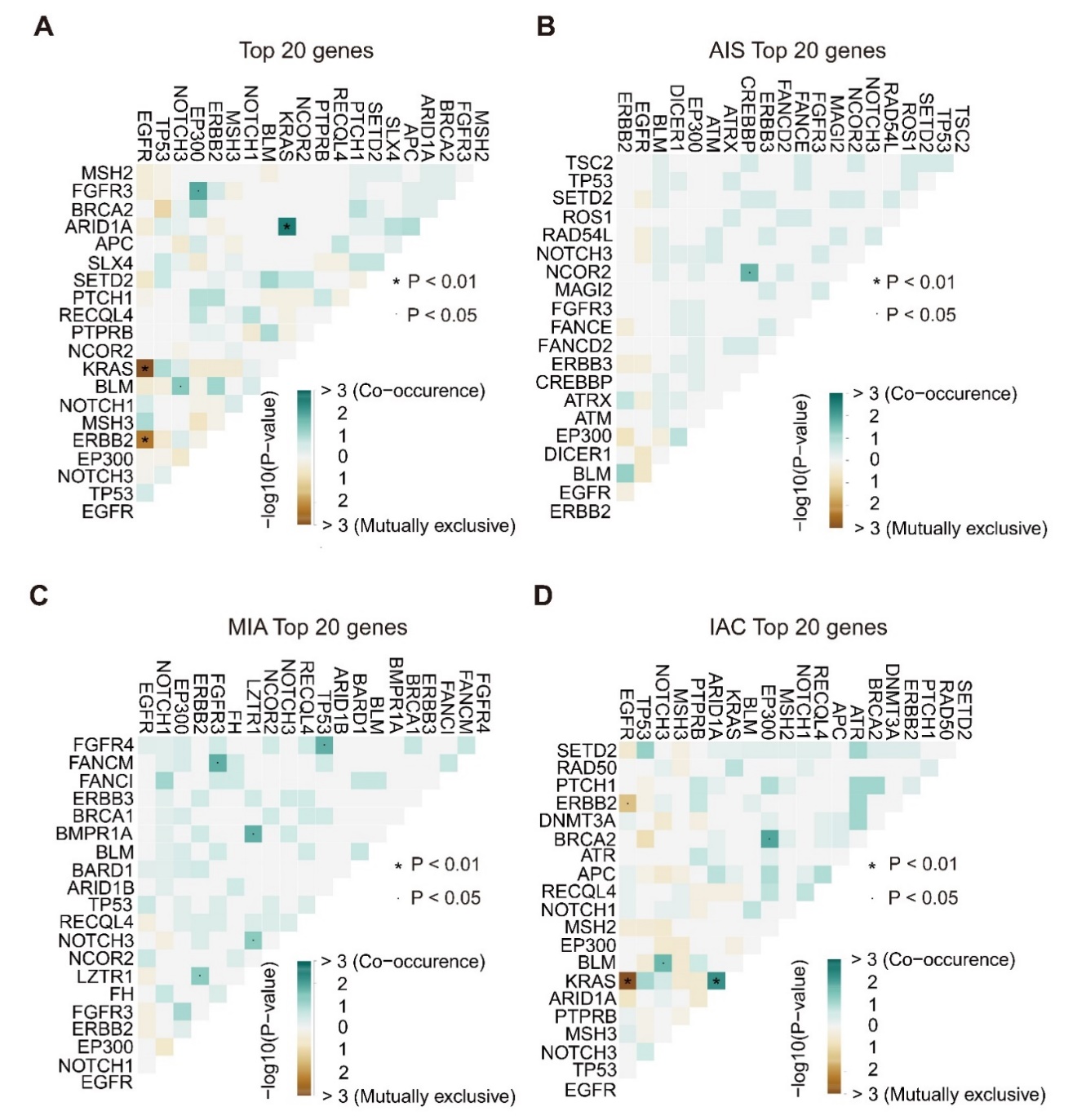


**Figure S2**. COME of mutations and mutational signatures in MPN. (A-D) Triangular matrixes exhibit the COME gene pairs. Green and brown refer to co-occurrence and mutual exclusivity, respectively (**p*<0.05). We identified three significant pairs of co-occurring driver genes: *ARID1A-KRAS* (*p*<0.01), *FGFR3-EP300* (*p*<0.05), and *BLM-TP53* (*p*<0.05), whereas *EGFR-KRAS* and *EGFR-ERBB2* displayed mutual exclusion. In subgroup analysis, only *FGFR3-EP300* (*p*<0.05) was identified in AIS. Five pairs of co-occurring genes (*LZTR1-ERBB2*, *FANCM-FGFR3*, *LZTR1-BMPR1A*, *LZTR1-NOTCH3*, *FGFR4-TP53,* all *p*<0.05) in MIA were identified. But no mutually exclusive driver gene was observed. In IAC subgroup, we identified three pairs of co-occurring genes: *KRAS-ARID1A* (*p*<0.01), *BLM-NOTCH3* (*p*<0.05), and *BRCA2-EP300* (*p*<0.05) and two pairs of mutually exclusive genes: *EGFR-KRAS* (*p*<0.01) and *EGFR-ERBB2* (*p*<0.05).


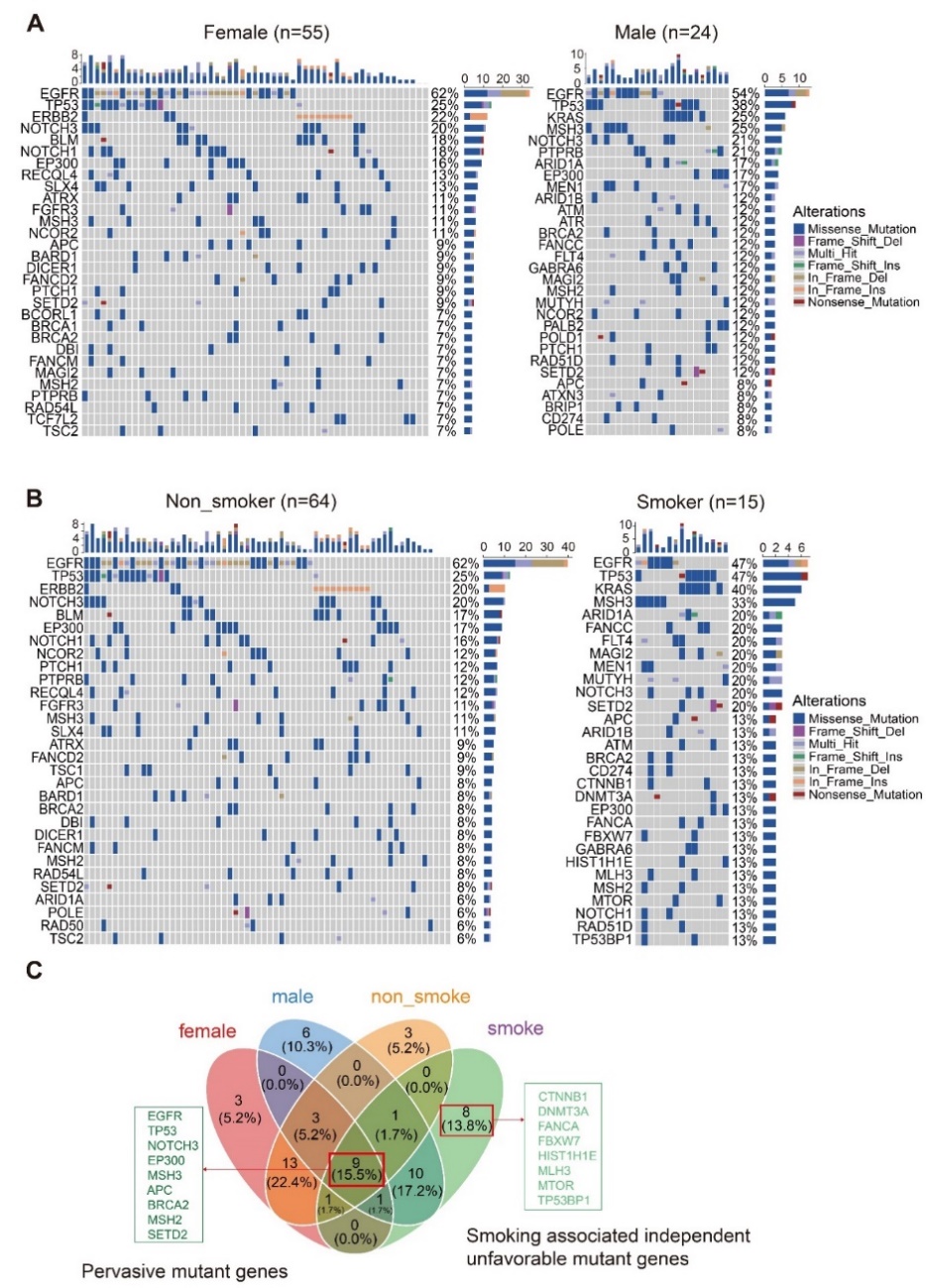


**Figure S3**. Mutations and their associations with sex and smoking status. (A-B) The mutation information of each gene in the female, male, smoker, and non-smoker panel, respectively. (C) Venn plot shows the shared mutant signatures among the four panels and the independent signature of the smoker in MPN.


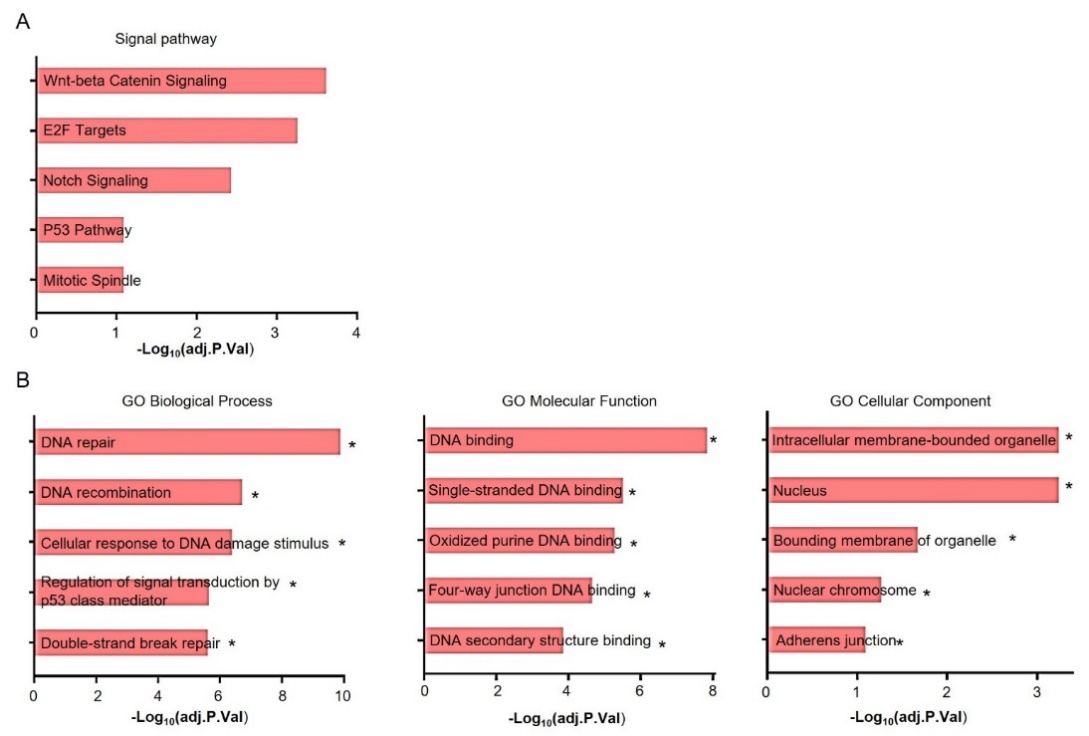


**Figure S4.** Signal pathway and gene ontology analysis. (A) Enrichment of MSigDB signal pathway analysis. (B) Enrichment of GO analysis (**p*<0.05).

**
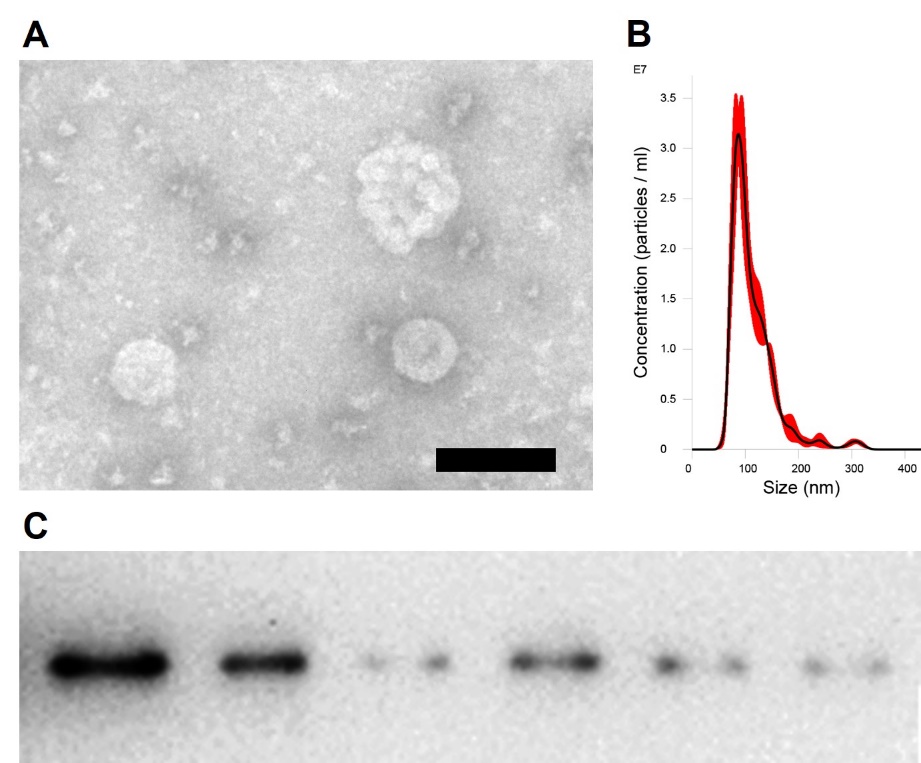
**

**Figure S5.** Characterization of patient derived EVs. (A) TEM image showing the morphology of EVs. Scale bar is 200 nm. Non-EV materials could be extracellular particles, aggregated serum proteins, or lipid droplets. (B) Size distribution of EVs. (C) Western blot of CD9 isolated from EVs derived from six patients.
